# Notes and News

**Published:** 1896-12-05

**Authors:** 


### Notes and News.

(Collected and Communicated.)

At a meeting of the Council of the Hospital Sunday Fund on the 26th ult., Sunday, June 20, being the Queen's Accession Day, was the date fixed for Hospital Sunday next year.

It has been decided to recommend that the Manchester Royal Infirmary shall be rebuilt on the present site. Thus, after prolonged discussion, this matter has very nearly been decided upon.

At the last quarterly general court of the Middlesex Hospital it was announced that the new cancer wards are so far in progress that plans have been prepared and estimates supplied. The money required is not all yet forthcoming, but contributions are arriving from many quarters. Mr. Bancroft's reading of the "Christmas Carol" produced the large sum of £300, and the trustees of Mr. Henry Delmar's estate have promised an annual subscription of £100 towards their maintenance when completed.

The fate of the £70,000 offered by the David Lewis Trustees to the people of Manchester to improve their hospital accommodation, which has so long hung in the balance, has at last been decided. The trustees, on learning that the committee of the St. Mary's Hospital were determinedly averse to the Stanley Grove site, have withdrawn their munificent offer, and therefore neither St. Mary's Hospital, the Southern Hospital, Owens College, nor the sick poor of Manchester will derive the looked-for benefit. The trustees are definitely of opinion that the Stanley Grove site is incomparably the best to be had in Manchester, and therefore they have altogether declined to meet the hospital on this point. £70,000 is a very large sum for any city, however wealthy, to reject, and nothing but the strongest motives, altogether independent of personal feeling, can warrant the refusal of the advantages which must have accrued from the acceptance of the gift.

Lord Kinnaird has accepted the position of trustee to the Dental Hospital of London, Leicester-square.

It is reported that all the victims of the present small-pox epidemic at Redruth were unvaccinated.

Mr. F. Treves presided at a dinner in aid of the National Dental Hospital and College, Great Portland Street, on the 27th ultimo. Mr. Treves commented on the useful work done by this hospital, and remarked that their aim was not so much to be great as to do good.

A correspondent suggests that by a little organisation it would be possible to obtain a large supply of cut flowers for the decoration of the wards of our hospitals by appointing agents at the railway termini to receive gifts from city men coming up from suburban homes where flowers are so plentiful. The suggestion is one to which we gladly give publicity. Everyone who has had experience of hospitals knows the great pleasure which their inmates derive from the presence of bright flowers and fresh greenery in the wards, but we also know how necessary it is that when such things are admitted they should be fresh and clean and absolutely free from all trace of decomposition. In hospitals, even more than in ordinary dwelling houses, it is necessary to ensure that such decorations should be frequently renewed. We, therefore, welcome any suggestion which may bring the great flower-growing public which dwells in the suburbs into relation with that less happy public which, being driven by its misfortunes to dwell in hospitals, hungers for fresh fruit and for the sight of flowers—bright, sweet, and redolent of the country.

A pending election to the staff of a provincial infirmary always brings forth a crop of grievances, and the case of the Bedford Infirmary is no exception to the rule. Among those made in this instance is one to the effect that, while the privilege of witnessing the operations at the infirmary ought to be open to all the medical men resident in the town, none except those who are partners of, or intimately associated with, the honorary surgeons are able to be present in consequence of there being no announcement of the operations to be performed. This is a point in hospital management which is of some importance. We do not think that in a small town it is possible without doing violence to the feelings of the patients and their relations to announce operations, and we have very little doubt that were such a course adopted the medical men in the town would be the first to attack the hospital surgeons for advertising themselves in so doing. The appointment of a regular operating day is, however, another matter. Every hospital should do this, and the staff of every hospital should welcome the outside medical men to witness the operations within its walls. To grant a right is another matter, and one to which the governors might fairly object, for even yet the possession of a medical qualification is not an absolute proof of reticence and amiability. This, however, we may say that there are few things which tend more to promote amity among the medical men of a country town, and to lead to good work among the surgeons of its hospital, than the custom of having a weekly consultation and operation day open to all the qualified men in the district. There will always be some operations which cannot be done in this public way; but the regular attendants will soon get the *entrée*, and those who only go occasionally, as to a show, are better kept out.

